# LncRNA-LET inhibits cell growth of clear cell renal cell carcinoma by regulating miR-373-3p

**DOI:** 10.1186/s12935-019-1008-6

**Published:** 2019-11-21

**Authors:** Zhuo Ye, Jiachen Duan, Lihui Wang, Yanli Ji, Baoping Qiao

**Affiliations:** 1 Department of Urology, The First Affiliated Hospital of Zhengzhou University, 1 East Jianshe Road, Zhengzhou, 450052 People’s Republic of China; 2 Department of Pathology and Pathophysiology, The Academy of Medical Sciences, Zhengzhou University, Zhengzhou, 450001 People’s Republic of China

**Keywords:** Clear cell renal cell carcinoma, LncRNA-LET, miR-373-3p, Cell cycle, Cell apoptosis

## Abstract

**Background:**

Clear cell renal cell carcinoma (ccRCC) is the most common renal cell carcinoma subtype with a poor prognosis. LncRNA-LET is a long non-coding RNA (lncRNA) that is down-regulated in ccRCC tissues. However, its role in ccRCC development and progress is unclear.

**Methods:**

LncRNA-LET expression was detected in ccRCC tissues and ccRCC cells using quantitative real-time PCR. The overexpression and knockdown experiments were performed in ccRCC cells and xenograft mouse model to evaluate role of lncRNA-LET. Cell cycle, apoptosis and JC-1 assays were conducted via flow cytometer. The protein levels were measured through western blot analysis and the interaction between lncRNA-LET and miR-373-3p was identified via luciferase reporter assay.

**Results:**

LncRNA-LET expression was lower in ccRCC tissues than that in the matched adjacent non-tumor tissues (n = 16). In vitro, lncRNA-LET overexpression induced cell cycle arrest, promoted apoptosis and impaired mitochondrial membrane potential, whereas its knockdown exerted opposite effects. Moreover, we noted that lncRNA-LET may act as a target for oncomiR miR-373-3p. In contrast to lncRNA-LET, miR-373-3p expression was higher in ccRCC tissues. The binding between lncRNA-LET and miR-373-3p was validated. Two downstream targets of miR-373-3p, Dickkopf-1 (DKK1) and tissue inhibitor of metalloproteinase-2 (TIMP2), were positively regulated by lncRNA-LET in ccRCC cells. MiR-373-3p mimics reduced lncRNA-LET-induced up-regulation of DKK1 and TIMP2 levels, and attenuated lncRNA-LET-mediated anti-tumor effects in ccRCC cells. In vivo, lncRNA-LET suppressed the growth of ccRCC xenograft tumors.

**Conclusion:**

These findings indicate that lncRNA-LET plays a tumor suppressive role in ccRCC by regulating miR-373-3p.

## Background

Renal cell carcinoma (RCC), as one of most frequent cancers worldwide, is a common lethal malignancy [1, 2]. RCC incidence and death rates are high, with 63,000 new cases and 14,000 deaths per year in United States [3]. Surgical resection and immunotherapy are currently being applied to treat patients with RCC [4–6]. RCC includes more than 10 histological and molecular subtypes [7], with clear cell RCC (ccRCC) as the most common subtype [8]. The detailed molecular mechanisms underlying ccRCC development remain elusive.

Non-coding RNAs (ncRNAs) are found to be important players in epigenetic regulation, especially long ncRNAs (> 200 nucleotides, lncRNAs) and microRNAs (< 22 nucleotides, miRNAs) [9–11]. Emerging evidence indicates that numerous dysregulated lncRNAs are involved in the development and progression of ccRCC [12, 13]. LncRNA-LET, as a recently identified lncRNA, was located at chromosome 15q24.1 [11, 14], and it plays a suppressive role in regulating cancer cell growth in malignancies, including esophageal squamous cell carcinoma and lung adenocarcinoma [14–16]. In ccRCC, the role that lncRNA-LET plays is unknown. Interestingly, lncRNA-LET expression is low in serum samples from patients with ccRCC [17], suggesting its involvement in the carcinogenesis of this cancer.

Previous studies have shown that lncRNA-LET has the potential to target miRNAs, thereby regulating the expression of miRNA targets to affect the process of human cancers [18, 19]. Although varied miRNAs may be regulated by lncRNA-LET, we here focused on miR-373-3p. Both tumor-promoting and anti-tumor effects of miR-373-3p have been reported before [20, 21]. An earlier study has revealed that miR-373-3p acts as an oncomiR in RCC [22]. By analyzing the sequence information of lncRNA-LET and miR-373-3p, we noted that lncRNA-LET contained a potential binding area for miR-373-3p. This study is thus performed to explore whether lncRNA-LET regulates the malignant behaviors of ccRCC cells by regulating miR-373-3p.

Herein, we explored the specific role of lncRNA-LET in regulating ccRCC growth in vitro and in vivo. LncRNA-LET overexpression or knockdown was performed in ccRCC cells. Meanwhile, the xenograft mouse model was constructed. We demonstrated that lncRNA-LET suppressed the growth of ccRCC cells. LncRNA-LET-induced cell cycle arrest and apoptosis in ccRCC cells were attenuated by miR-373-3p mimics.

## Materials and methods

### Patients and tissues

The human ccRCC and matched adjacent non-tumor tissues were obtained from 16 ccRCC patients from the First Affiliated Hospital of Zhengzhou University during September 2018–November 2018. Each patient provided an informed consent prior to specimen collection. This study was approved by the Ethics Committee of the First Affiliated Hospital of Zhengzhou University, and conferred to Declaration of Helsinki.

### Cell culture and transfection

The ccRCC cell lines (Caki-1, 786-O, 769-P) and 293T cell line were purchased from Procell Life Science & Technology Co,. Ltd. (Wuhan, China). Caki-1 cells were cultured in McCoy’s 5A medium (Procell Life Science & Technology Co,. Ltd) containing 10% fetal bovine serum (FBS; Biological Industries, Kibbutz Beit-Haemek, Israel), 786-O and 769-P cells were cultured in RPMI-1640 medium (Procell Life Science & Technology Co,. Ltd.) supplemented with 10% FBS, and 293T cells were grown in DMEM medium (Procell Life Science & Technology Co,. Ltd.) containing 10% FBS in an incubator at 37 °C and 5% CO_2_. 786-O cells were transiently transfected with lncRNA-LET overexpression (lncRNA-LET OV), negative control (OV NC) vector, miR-373-3p inhibitor or negative control inhibitor (inhibitor NC), whereas 769-P cells were transiently transfected with lncRNA-LET siRNA, siRNA NC, miR-373-3p mimics or negative control mimics (mimics NC). In addition, 786-O cells were co-transfected with lncRNA-LET OV and miR-373-3p mimics or mimics NC. The miR-373-3p inhibitor, inhibitor NC, miR-373-3p mimics and mimics NC were purchased from JTSBIO (Wuhan, China). Further, 786-O cells stably transfected with lncRNA-LET overexpression (lncRNA-LET) or empty control (EV), 769-P cells stably transfected with lncRNA-LET knockdown (lncRNA-LET shRNA) or control (shRNA Ctrl) were established by selecting cells with 200 μg/ml or 300 μg/ml G418.

### Plasmid construction

The pcDNA3.1 and pRNAH1.1 vectors were purchased from GenScript (Nanjing, China). To overexpress lncRNA-LET, pcDNA3.1-lncRNA-LET was constructed by GenScript (Nanjing, China). The lncRNA-LET siRNAs and siRNA NC were purchased from JTSBIO (Wuhan, China). The shRNA targeting lncRNA-LET was designed and synthesized, and then cloned into pRNAH1.1 vector to generate the shRNA against lncRNA-LET (lncRNA-LET shRNA). The sequences of lncRNA-LET siRNA-1, lncRNA-LET siRNA-2 and lncRNA-LET shRNA were listed in Table 1.

**Table 1:** Sequence information

Name	Sequences
lncRNA-LET siRNA-1	Sense: 5′-GUCUGAUGUAUCCACCCAUTT-3′
	Antisense: 5′-AUGGGUGGAUACAUCAGACTT-3′
lncRNA-LET siRNA-2	Sense: 5′-GUGCAUGUGGUAGGUUAGATT-3′
	Antisense: 5′-UCUAACCUACCACAUGCACTT-3′
lncRNA-LET shRNA	Sense: 5′-GATCCGGTCTGATGTATCCACCCATTTCAAGAGAATGGGTGGATACATCAGACTTTTTA-3′
	Antisense: 5′-AGCTTAAAAAGTCTGATGTATCCACCCATTCTCTTGAAATGGGTGGATACATCAGACCG-3′

### Quantitative real-time PCR

Total RNAs were extracted using RL reagent (BioTeke, China). The mRNA was reversely transcribed into complementary DNA with M-MLV Reverse Transcriptase (Takara, China). The expression levels of lncRNA-LET and miR-373-3p were detected via SYBR Green (BioTeke, China). β-actin and U6 were applied as internal controls. The relative expression levels were calculated with the 2^−ΔΔCt^ method. The primers were shown in Table 2.

**Table 2:** Primers for quantitative real-time PCR

Gene name	Primer sequences
lncRNA-LET	Forward: 5′-AGCGTTTACTTCGTTGTTGT-3′
	Reverse: 5′-CAGAATGGAAATACTGGAGC-3′
β-actin	Forward: 5′-CACTGTGCCCATCTACGAGG-3′
	Reverse: 5′-TAATGTCACGCACGATTTCC-3′
miR-373-3p	Forward: 5′-GGCGGAAGTGCTTCGATTTT-3′
	Reverse: 5′-GTGCAGGGTCCGAGGTATTC-3′
U6	Forward: 5′-GCTTCGGCAGCACATATACT-3′
	Reverse: 5′-GTGCAGGGTCCGAGGTATTC-3′

### Cell cycle analysis

The 786-O and 769-P cells were firstly cultured in RPMI-1640 medium supplemented with 10% FBS, respectively. Then, the same batch of cells (4 × 10^5^/well) were seeded onto 6-well plates and cultured in RPMI-1640 medium containing 10% FBS. 786-O cells were transiently transfected with lncRNA-LET OV or OV NC vector, or co-transfected with lncRNA-LET OV and miR-373-3p mimics or mimics NC. The 769-P cells were transiently transfected with lncRNA-LET siRNA or siRNA NC. After 48 h, cells were collected and fixed in ice-cold 70% ethanol for 12 h at 4 °C, and then incubated with 25 μl propidium iodide (PI) and 10 μl RNase A (Beyotime, China) for 30 min at 37 °C in the dark. Cell cycle distribution was analyzed using flow cytometer.

### EdU assay

Cells were cultured with cell medium containing a final concentration of 10 μM EdU (Keygen, China) for 2 h. They were then fixed in 4% paraformaldehyde for 15 min, and incubated with 0.5% Triton X-100 for 20 min at room temperature. Cells were subsequently washed twice with PBS containing 3% BSA and then reacted with Click-iT for 30 min. The nuclei were stained with Hoechst 33342 (1:2000, Keygen, China) for 15 min. Finally, the images were captured under fluorescence microscopy and the EdU-positive cells were calculated.

### Western blot analysis

Total proteins were obtained using RIPA buffer (Beyotime, China), and mitochondrial proteins were extracted with Mitochondrial Protein Extraction Kit (BOSTER, China). Then, protein concentrations were determined via a BCA Protein Assay Kit (Beyotime, China). Proteins were separated through SDS-PAGE and transferred to PVDF membranes. After blocking in 5% BSA, the membranes were subsequently incubated with primary antibodies, including Cyclin D1 (1:500; #2978, CST, USA), Cyclin E (1:500; #20808, CST, USA), Bax (1:5000; 50599-2-Ig, Proteintech, China), Bcl-2 (1:500; 12789-1-AP, Proteintech, China), Cytochrome *C* (1:5000; ab133504, Abcam, UK), Dickkopf-1 (DKK1) (1:1000; 21112-1-AP, Proteintech, China), tissue inhibitor of metalloproteinase-2 (TIMP2) (1:500; A1558, Abclonal, China), and β-actin (1:2000; 60008-1-Ig, Proteintech, China) overnight at 4 °C. Afterwards, the membranes were incubated with the secondary antibody (1:10,000; SA00001-1 or SA00001-2, Proteintech, China) for 40 min at 37 °C. Signals were detected with enhanced chemiluminescence (7 Sea biotech, China).

### Cell apoptosis detection

Cells were collected and centrifuged at 1000*g* for 5 min. Then, the cells in 195 μl binding buffer were incubated with 5 μl AnnexinV-FITC and 10 μl PI for 15 min at room temperature in the dark according to the manufacturer’s instruction (Beyotime, China). Cell apoptosis was analyzed by flow cytometer.

### Caspase activity assay

The activities of caspase-3 and caspase-9 were analyzed with corresponding Caspase Assay Kits (Beyotime or Solarbio, China). Briefly, proteins were extracted from cells and then qualified with Bradford Protein Assay Kit (Beyotime, China). Subsequently, samples were incubated with the caspase substrate for 24 h at 37 °C. The absorbance was determined at 405 nm.

### JC-1 assay

Cells were obtained and centrifuged at 550*g* for 5 min. Then, the cells were resuspended in 500 μl JC-1 staining working solution (Beyotime, China). After incubation for 20 min in the incubator at 37 °C, cells were centrifuged at 600*g* for 5 min and washed twice with 1× JC-1 staining buffer, and resuspended with 500 μl 1× JC-1 staining buffer. JC-1 aggregate was measured via the flow cytometer.

### Hematoxylin–eosin (HE) staining

The tumor tissues were fixed with 4% paraformaldehyde, embedded with paraffin and then cut into 5-µm sections. Afterwards, the sections were deparaffinized and rehydrated before being stained with hematoxylin (Solarbio, China) and eosin (Sangon, China). The staining was visualized under a microscope.

### TUNEL staining

The tumor tissues were fixed with 4% paraformaldehyde and 5-µm sections were embedded in paraffin, followed by deparaffinization and rehydration. The TUNEL-positive cells were labeled by Label Solution with Enzyme solution for 60 min at 37 °C in the dark, and then these sections were incubated with converter-peroxidase (POD) according to the manufacturer’s protocol. Afterwards, hematoxylin (Solarbio, China) was used for the counterstaining of cell nuclei. The analysis of apoptotic cells was conducted and images were taken under a microscope.

### Immunofluorescence analysis

Cells were fixed in 4% paraformaldehyde for 15 min and incubated with 0.1% Triton X-100 (Beyotime, China) for 30 min. Additionally, tumor tissues were fixed in 4% paraformaldehyde, embedded with paraffin and cut into 5-µm sections. Then, the sections were incubated with goat serum to block nonspecific binding. The sections were subsequently incubated with anti-Ki67 antibody (1:50, Proteintech, China) or anti-Cytochrome *C* antibody (1:100, proteintech, China) overnight at 4 °C. After washing thrice with PBS, the sections were incubated with Cy3 goat anti-rabbit IgG (1:200, Beyotime, China) and counterstained with DAPI (Biosharp, China). The results were analyzed under a fluorescence microscope.

### Luciferase reporter assay

293 T cells were seeded onto 12-well plates. The partial lncRNA-LET sequences containing wild-type (WT) and mutant (MUT) binding sites for miR-373-3p were synthesized and subcloned into pmirGLO luciferase reporter vectors. The 293 T cells were transfected with the luciferase reporter constructs together with miR-373-3p mimics or mimics NC with Lipofectamine 2000. After a 48-h incubation, the transfected cells were collected and the luciferase activity analysis was conducted.

### In viv*o* xenograft mouse model

Male 5-week-old BALB/c nude mice were obtained from BEIJING HFK BIOSCIENCE Co., LTD (China). All animal experiments were conducted according to the Guideline for the Care and Use of Laboratory Animals and approved by the Ethics Committee of the First Affiliated Hospital of Zhengzhou University. Mice acclimated for 1 week and then randomly assigned to four groups (n = 6/group): EV group, lncRNA-LET group, shRNA Ctrl group, lncRNA-LET shRNA group. 5 × 10^6^ cells stably expressing EV, lncRNA-LET, shRNA Ctrl or lncRNA-LET shRNA vectors were subcutaneously injected in the right fore-flank of each nude mouse. Then, the size of the tumor was recorded every 3 days for 21 days. Finally, the mice were sacrificed and tumor tissues were photographed. The tumor volume was calculated with the equation volume (mm^3^) = length × width^2^/2.

### Statistical analysis

All date were analyzed with GraphPad Prism version 7.0 and presented as mean ± SD. The two-tailed paired and unpaired Student’s t-test was used to test for significant differences between two groups. One-way ANOVA analysis followed by Tukey’s test was used to analyze the multi-sample analysis. p value less than 0.05 was considered statistically significant.

## Results

### LncRNA-LET expression is down-regulated in ccRCC tissues

**Fig. 1:**
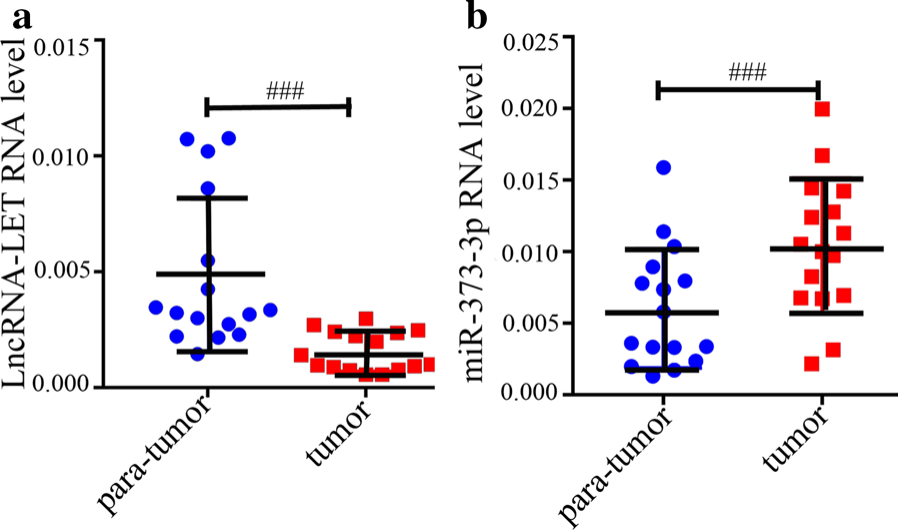
LncRNA-LET expression is down-regulated in ccRCC tissues. The expression levels of LncRNA-LET (**a**) and miR-373-3p (**b**) were detected using quantitative real-time PCR in ccRCC tissues and matched adjacent non-tumor tissues. n = 16, ^###^p<0.001. *ccRCC* clear cell renal cell carcinoma

To examine the clinical significances of lncRNA-LET in ccRCC tissues, we conducted quantitative real-time PCR. The lncRNA-LET expression level was significantly decreased in ccRCC tissues compared with matched adjacent non-tumor tissues (Fig. 1a). On the contrary, miR-373-3p expression was higher in ccRCC tissues (Fig. 1b).

### LncRNA-LET arrests cell cycle at G1 stage in ccRCC cells

**Fig. 2:**
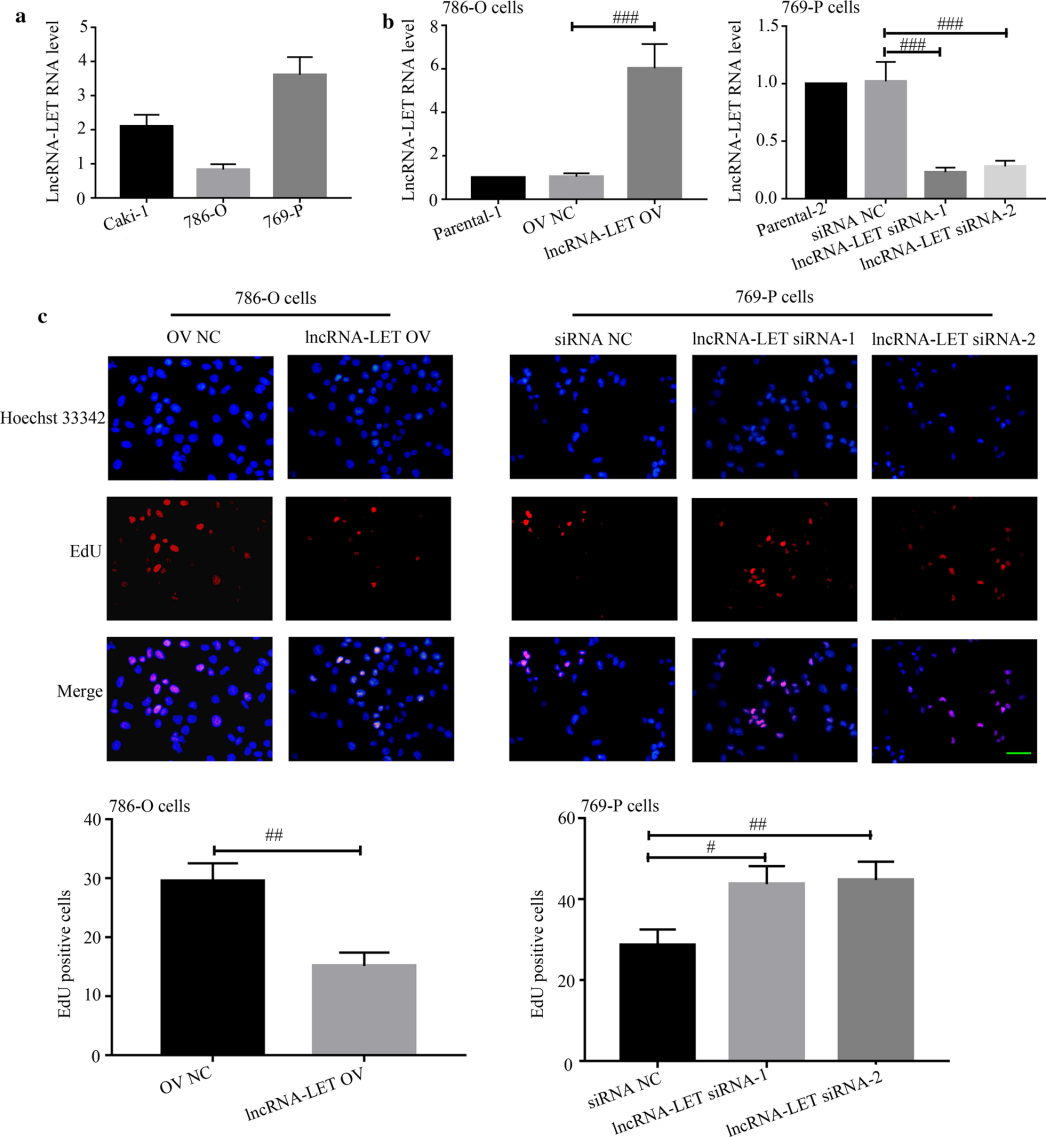
LncRNA-LET arrests cell proliferation in ccRCC cells. **a** LncRNA-LET level was measured in Caki-1, 786-O, 769-P cells by quantitative real-time PCR. **b** The level of lncRNA-LET was assayed in lncRNA-LET overexpression 786-O cells or lncRNA-LET knockdown 769-P cells via quantitative real-time PCR. **c** Cell proliferation was detected via EdU staining. Scale bars, 50 μm. ^#^p < 0.05, ^##^p < 0.01, ^###^p < 0.001. *ccRCC* clear cell renal cell carcinoma

**Fig. 3:**
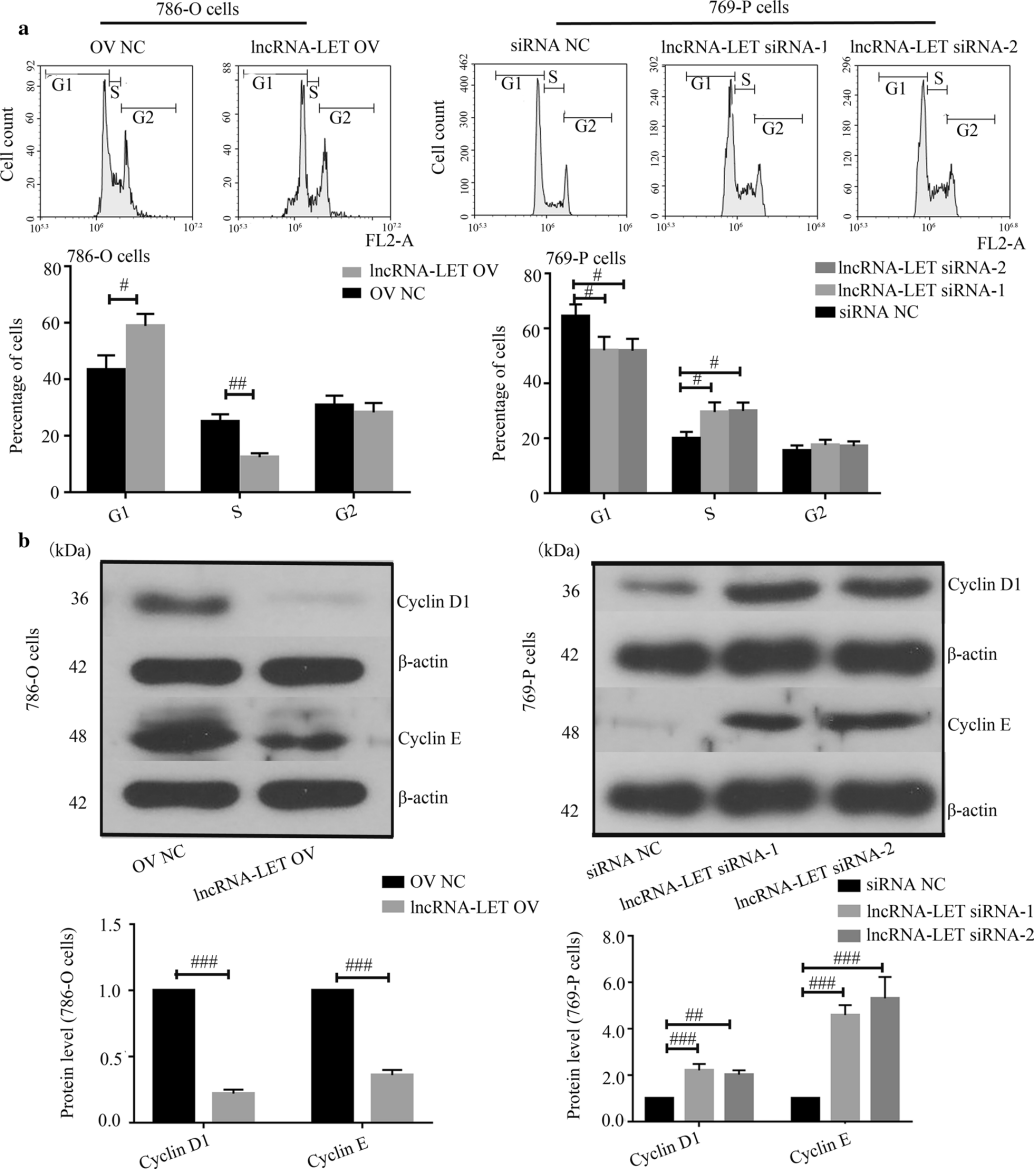
LncRNA-LET arrests cell cycle in ccRCC cells. **a** The flow cytometry was used to analyze cell cycle phase distribution. **b** The Cyclin D1 and Cyclin E protein levels were analyzed with western blot analysis. ^##^p < 0.01, ^###^p < 0.001. *ccRCC* clear cell renal cell carcinoma

The basal expression levels of lncRNA-LET in three ccRCC cancer cell lines were first determined via quantitative real-time PCR. The lowest and highest lncRNA-LET expression were observed in 786-O cells and 769-P cells, respectively (Fig. 2a). Next, lncRNA-LET overexpression plasmid was transfected into 786-O cells, while two lncRNA-LET siRNAs were transfected into 769-P cells. The overexpression and knockdown efficiencies were confirmed via quantitative real-time PCR in these two cell lines (Fig. 2b). EdU incorporation assays demonstrated that lncRNA-LET inhibited ccRCC cell proliferation (Fig. 2c). Cell cycle progression detection revealed that lncRNA-LET overexpression caused a dramatic accumulation in G1-phase and reduction in S-phase of 786-O cells, whereas lncRNA-LET silencing accelerated cell cycle of 769-P cells to S-phase (Fig. 3a). Moreover, lncRNA-LET overexpression down-regulated the expression of Cyclins D1 and E in 786-O cells, while lncRNA-LET knockdown up-regulated their expression in 769-P cells (Fig. 3b). These findings suggest that lncRNA-LET suppresses the proliferation and arrests cell cycle progress of ccRCC cells.

### LncRNA-LET promotes cell apoptosis in ccRCC cells

**Fig. 4:**
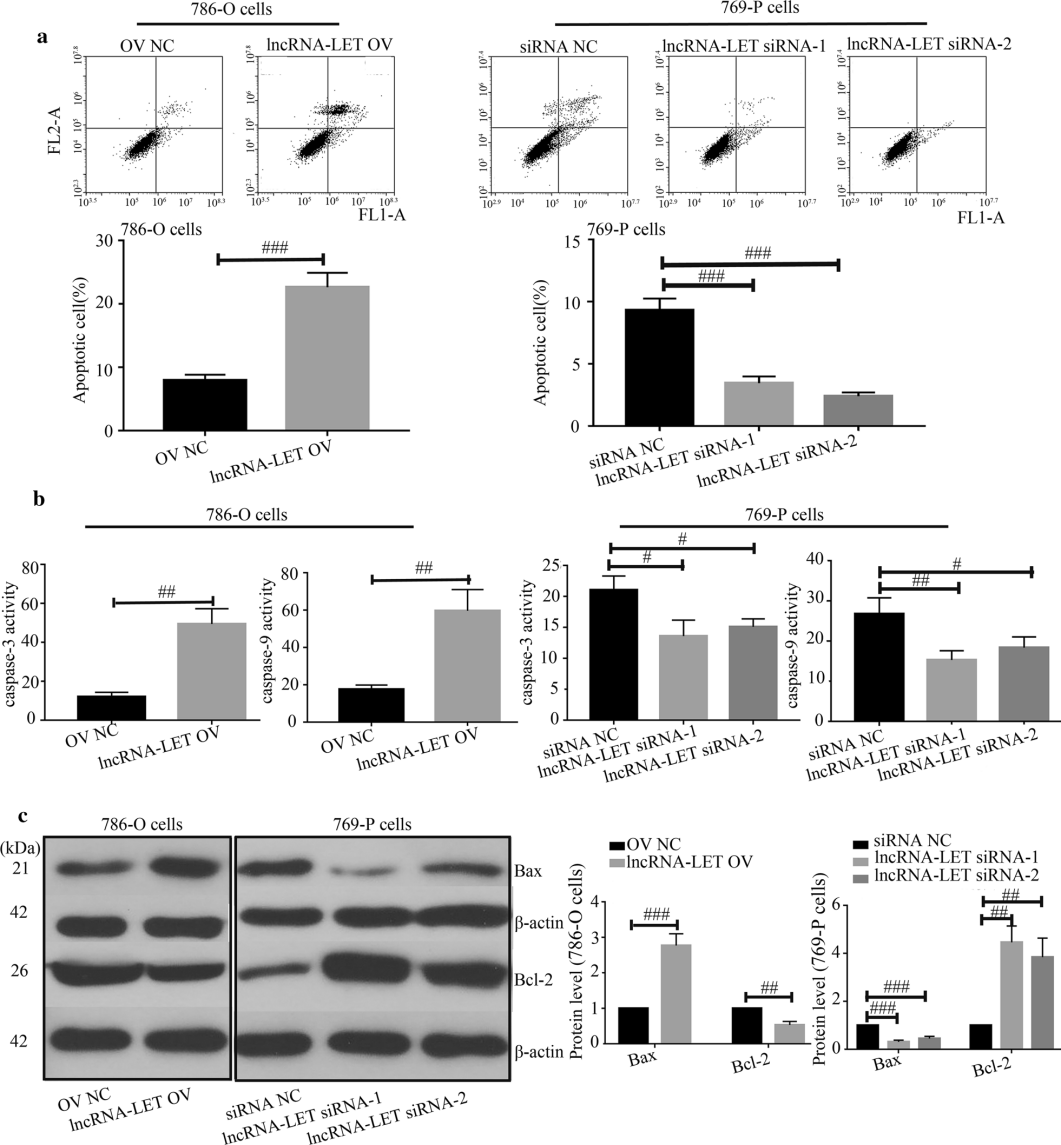
LncRNA-LET promotes cell apoptosis in ccRCC cells. **a** Cell apoptosis was studied by flow cytometry. **b** The caspase-3 and caspase-9 activities were detected through corresponding caspase assay kits. **c** Western blot analysis was used to determine the Bax and Bcl-2 protein levels. ^#^p < 0.05, ^##^p < 0.01, ^###^p < 0.001. *ccRCC* clear cell renal cell carcinoma

**Fig. 5:**
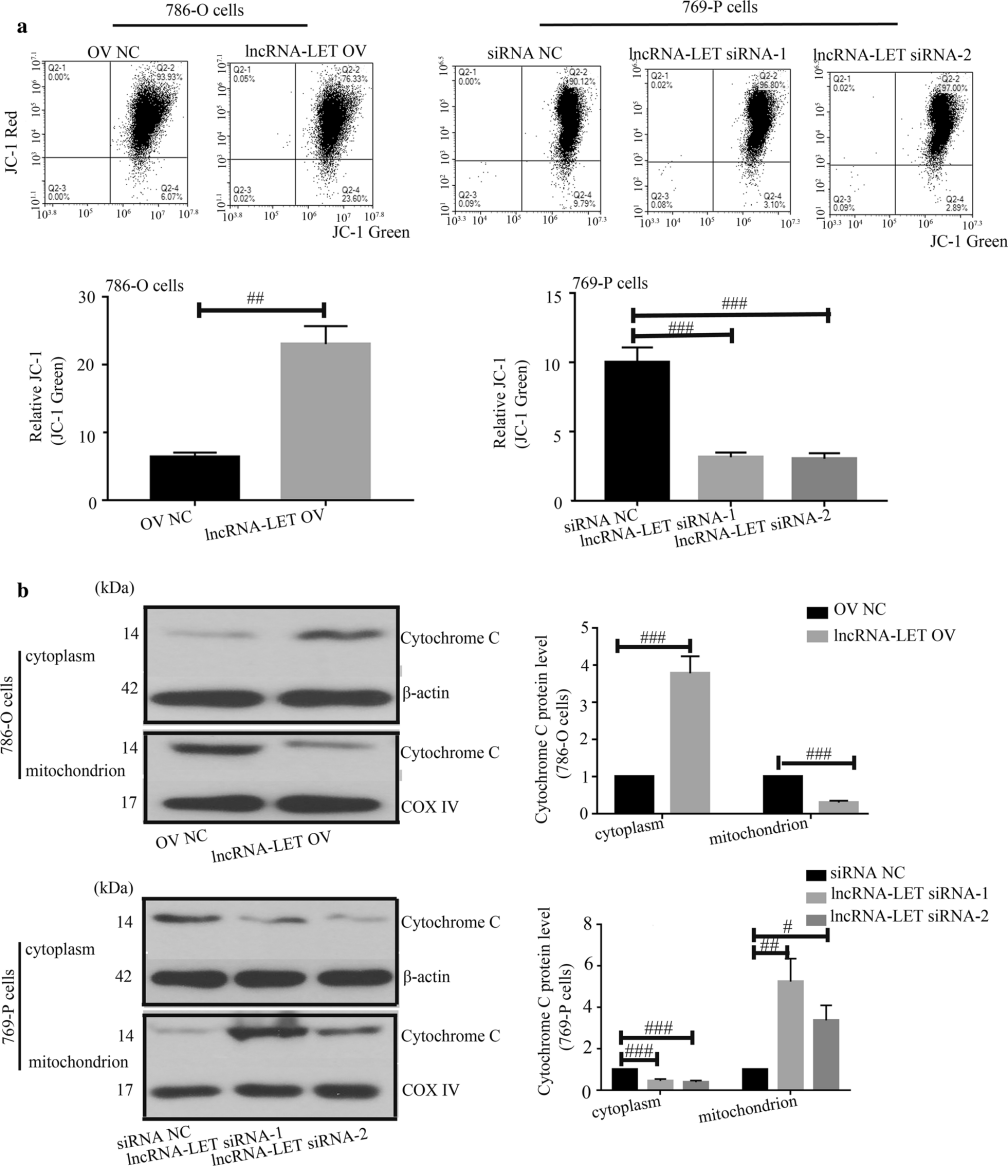
LncRNA-LET promotes mitochondrial membrane potential and cytochrome *C* release in ccRCC cells. **a** The ratio of green/monomeric forms of JC-1 dye was calculated with flow cytometry. **b** Cytochrome *C* protein level in cytoplasm or mitochondrion was examined using western blot analysis. ^#^p < 0.05, ^##^p < 0.01, ^###^p < 0.001. *ccRCC* clear cell renal cell carcinoma

**Fig. 6:**
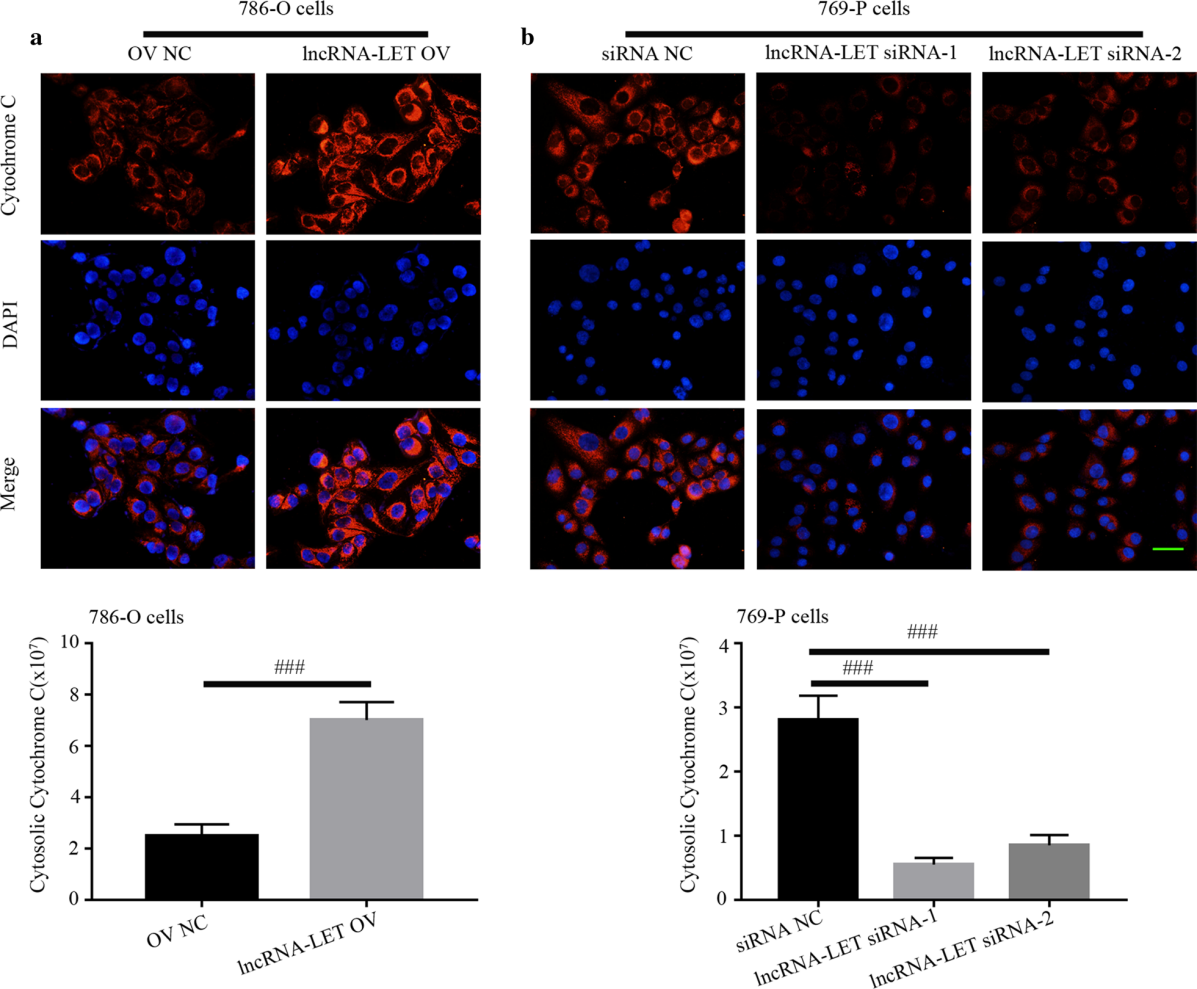
Effect of lncRNA-LET on Cytochrome *C* translocation in ccRCC cells. **a**, **b** Immunofluorescence detected the cytosolic translocation of Cytochrome *C*. Scale bar, 50 μm. ^###^p < 0.001. *ccRCC* clear cell renal cell carcinoma

Results from flow cytometry indicated that lncRNA-LET overexpression significantly promoted cell apoptosis while lncRNA-LET silencing suppressed cell apoptosis (Fig. 4a). Meanwhile, lncRNA-LET increased caspase-3 and caspase-9 activities (Fig. 4b), up-regulated Bax expression and reduced Bcl-2 expression (Fig. 4c) in 786-O cells. Data from JC-1 assay illustrated that lncRNA-LET increased the ratio of green/monomeric forms of JC-1 in ccRCC cells (Fig. 5a). Further western blot analysis confirmed that lncRNA-LET led to the release of Cytochrome *C* from mitochondria (Fig. 5b). Immunofluorescence also showed that lncRNA-LET overexpression significantly facilitated cytosolic translocation of Cytochrome *C* and lncRNA-LET silencing inhibited its translocation in ccRCC cells (Fig. 6a, b). The data reveal that lncRNA-LET promotes cell apoptosis in ccRCC cells possibly.

### LncRNA-LET targets miR-373-3p to regulate ccRCC cell growth

**Fig. 7:**
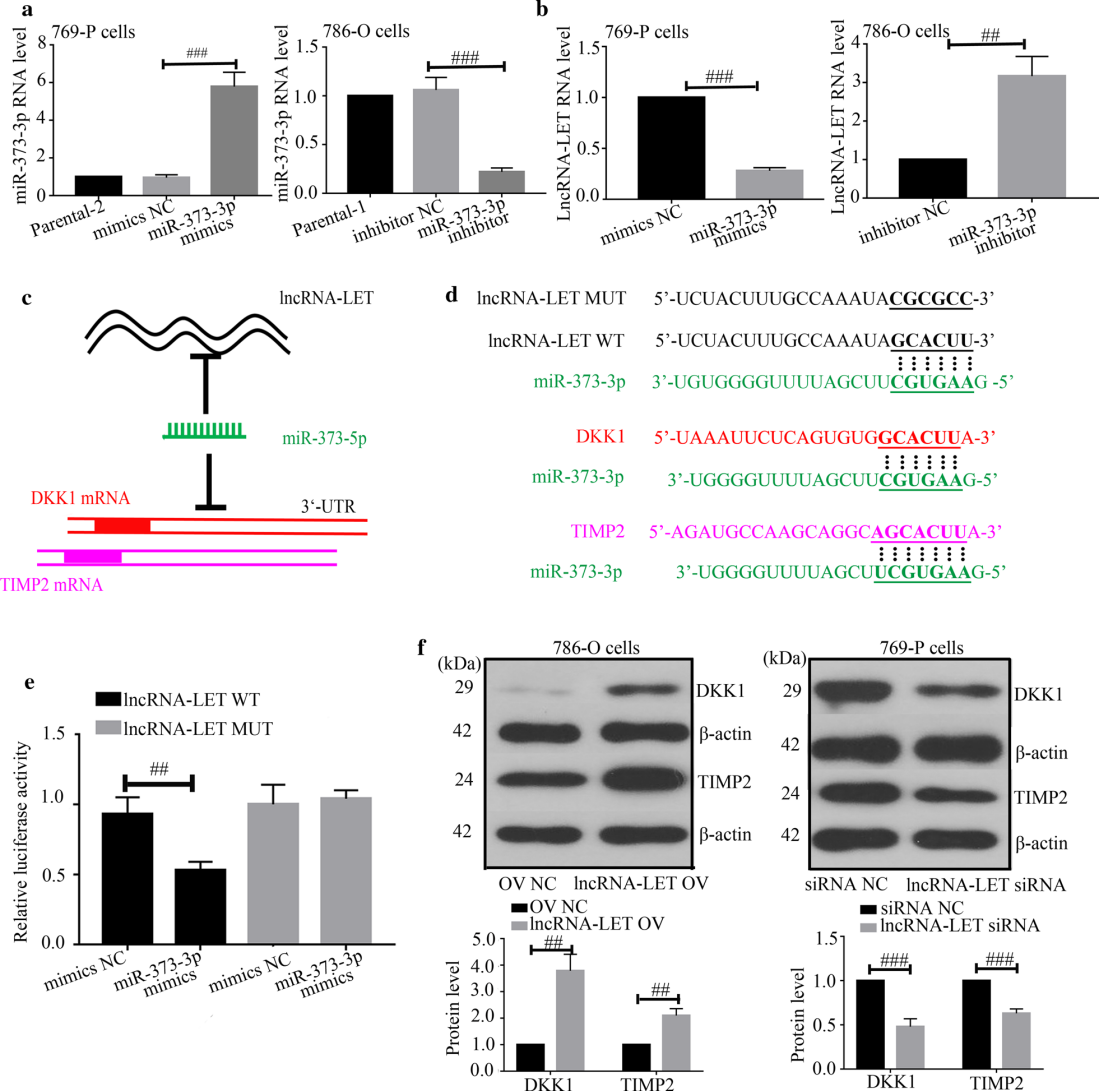
LncRNA-LET is involved in cell growth of ccRCC cells by targeting miR-373-3p. **a** MiR-373-3p level was assayed in ccRCC cells with miR-373-3p mimics or inhibitor via quantitative real-time PCR. **b** LncRNA-LET level was assayed in miR-373-3p mimics or inhibitor ccRCC cells via quantitative real-time PCR. **c** Schematic diagram: lncRNA-LET (black) function as a target of miR-373-3p (green). MiR-373-3p was indicated to bind with DKK1 (red) and TIMP2 (burgandy). ORF was filled with rectangles. **d** The binding sites between lncRNA-LET and miR-373-3p, miR-373-3p and DKK1, miR-373-3p and TIMP2 were predicted. **e** The relationship between lncRNA-LET and miR-373-3p was demonstrated through luciferase reporter assay. **f** DKK1 and TIMP2 protein levels were examined with western blot analysis. ^##^p < 0.01, ^###^p < 0.001. *ccRCC* clear cell renal cell carcinoma, *DKK1* Dickkopf-1, *TIMP2* tissue inhibitor of metalloproteinase-2, *mimics NC* negative control mimics

**Fig. 8:**
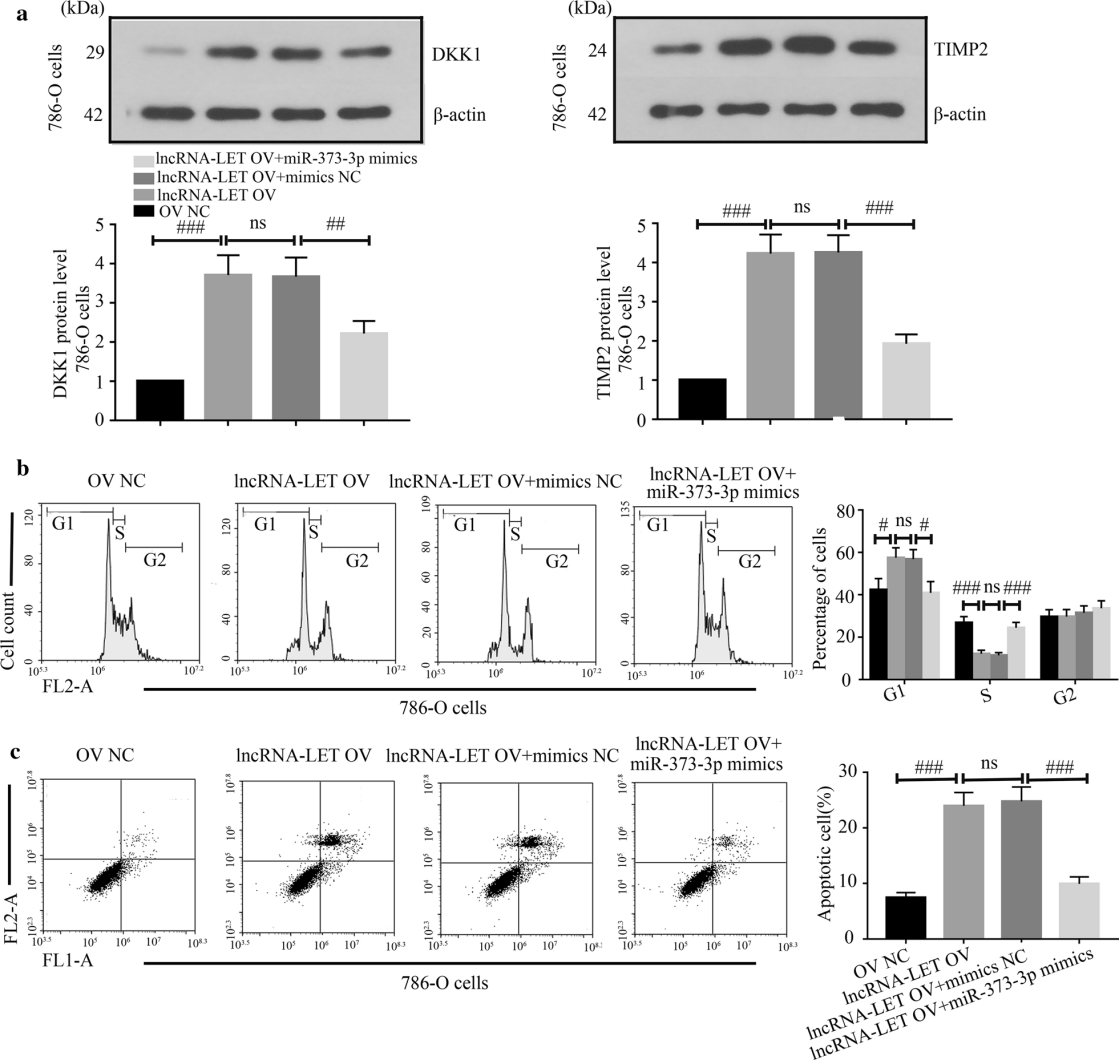
MiR-373-3p mimics alleviate the effects of lncRNA-LET overexpression on cell cycle and apoptosis. **a** Western blot analysis was used to measure the DKK1 and TIMP2 protein levels. Cell cycle phase distribution (**b**) and cell apoptosis (**c**) were analyzed through flow cytometry. ^##^p < 0.01, ^###^p < 0.001. ns, *ccRCC* clear cell renal cell carcinoma, *mimics NC* negative control mimics

We further explored the mechanism underlying the role of lncRNA-LET in ccRCC. We hypothesized that lncRNA-LET bound to miR-373-3p to regulate development of ccRCC. As shown in Fig. 1b, the miR-373-3p expression level was increased in ccRCC tissues. Then, we carried out miR-373-3p overexpression or knockdown in ccRCC cells (Fig. 7a). Quantitative real-time PCR revealed that miR-373-3p mimics down-regulated lncRNA-LET expression, whereas miR-373-3p inhibitor up-regulated the lncRNA-LET expression level (Fig. 7b). MiR-373-3p was predicted to bind to lncRNA-LET (Fig. 7c, d). Luciferase report assay confirmed the interaction between lncRNA-LET and miR-373-3p (Fig. 7e). Further, we found that lncRNA-LET positively regulated DKK1 and TIMP2 expression in ccRCC cells (Fig. 7f). However, miR-373-3p mimics reduced the DKK1 and TIMP2 expression caused by lncRNA-LET (Fig. 8a). Additionally, miR-373-3p mimics alleviated the effects of lncRNA-LET overexpression on cell cycle and apoptosis (Fig. 8b, c). These data indicate that lncRNA-LET inhibits cell growth of ccRCC cells through targeting miR-373-3p.

### LncRNA-LET inhibits tumor growth in vivo

**Fig. 9:**
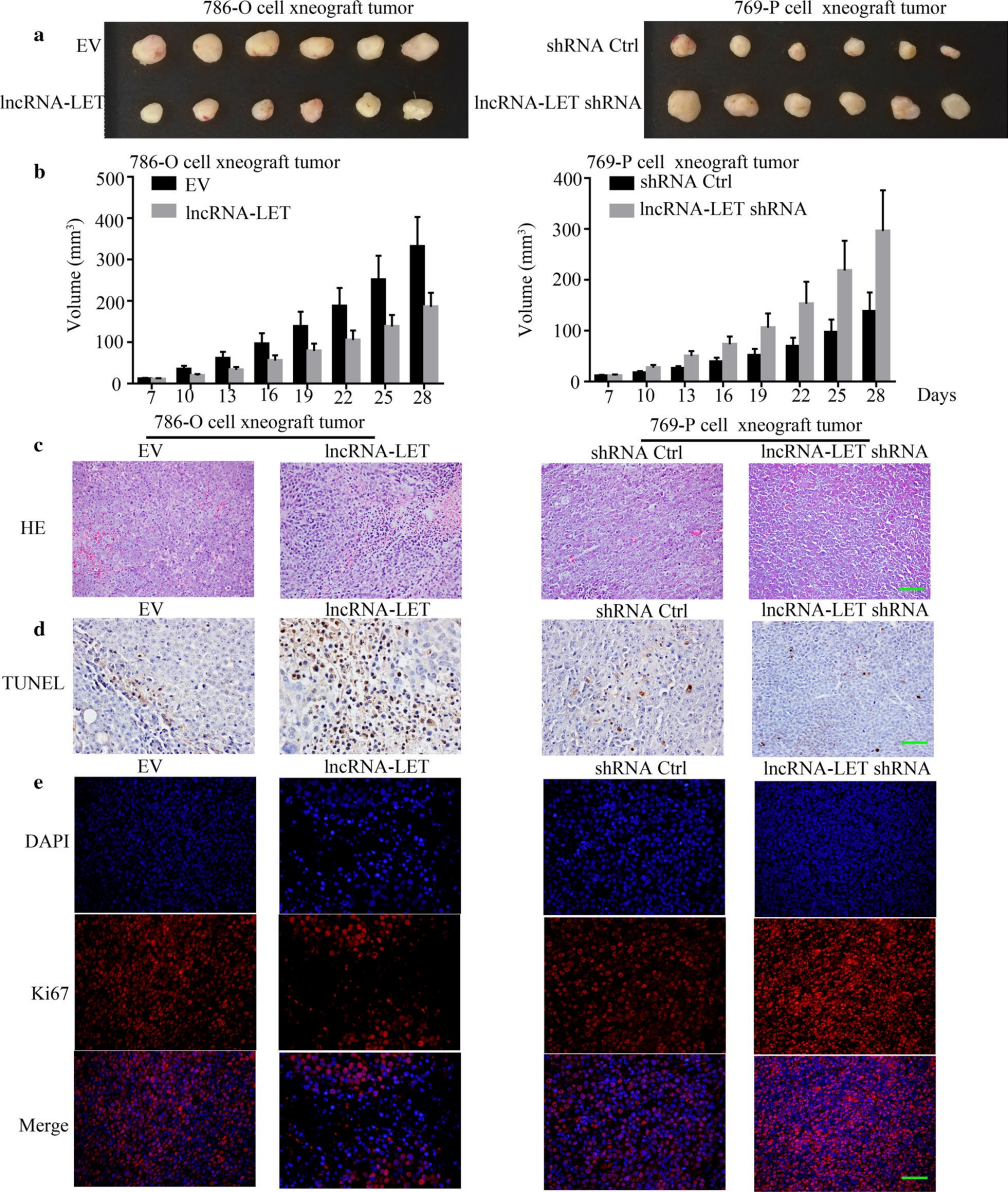
LncRNA-LET inhibits tumor growth in vivo. 786-O cell xenograft tumors stably transfected with lncRNA-LET or EV vectors, as well as 769-P cell xenograft tumors stably transfected with lncRNA-LET shRNA or shRNA Ctrl vectors were obtained. The size of the tumor was then recorded every 3 days for 21 days. Finally, the mice were sacrificed, and tumor tissues were (**a**) took pictures. **b** The volume of tumor tissues were measured by caliper and then calculated. **c** Cell apoptosis and necrosis from tumor tissues was analyzed using HE staining. Scale bars, 100 μm. **d** Cell apoptosis in tumor tissues was detected through TUNEL staining. Scale bars, 50 μm. **e** A representative Ki67 immunofluorescence in tumor tissues was explored. Scale bars, 50 μm. n = 6, ^##^p < 0.01. *ccRCC* clear cell renal cell carcinoma, *lncRNA-LET* lncRNA-LET overexpression, *EV* lncRNA-LET overexpression matched control, *lncRNA-LET shRNA* lncRNA-LET knockdown, *shRNA Ctrl* lncRNA-LET knockdown control

In order to evaluate the function of lncRNA-LET in tumor growth in vivo, we stably overexpressed lncRNA-LET in 786-O cells or knocked lncRNA-LET down in 769-P cells. These stably transfected cells were subcutaneously injected into nude mice. LncRNA-LET led to reduction of tumor volume (Fig. 9a, b). Further HE staining, TUNEL staining and Ki67 immunostaining results showed that lncRNA-LET increased apoptosis, and suppressed cell proliferation (Fig. 9c–e). These results indicate that lncRNA-LET functions similarly in vivo and in vitro.

## Discussion

In this study, low lncRNA-LET was found in ccRCC tissues compared with matched adjacent non-tumor tissues. LncRNA-LET induced cell cycle arrest and apoptosis of ccRCC in vitro and inhibited the growth of xenografts in vivo. Further study showed lncRNA-LET performed its role in ccRCC by targeting miR-373-3p.

LncRNAs are reported to regulate various biological processes. They can contribute to the tumor progression or act as tumor-suppressors in ccRCC [12, 23–25]. It was reported that the low lncRNA-LET level was correlated to the poor prognosis of patients with lung cancer or gastric cancer [26, 27]. Wu et al. [17] showed a low lncRNA-LET level in the serum of ccRCC patients and regarded it as highly indicative of ccRCC diagnosis. In this study, we confirmed a low lncRNA-LET level in ccRCC tissues, which was consistent with the report of Wu et al. However, the role of lncRNA-LET in the growth of ccRCC is still unclear and needs further exploration.

In our study, lncRNA-LET inhibited the growth of ccRCC, both in vitro and in vivo. Cell cycle contributes to the growth of cells, and cyclins are key drivers of cell cycle. The growth-inhibitory effect of lncRNA-LET was accompanied with cell cycle arrest, which was further confirmed by declines in cyclins. Besides, lncRNA-LET also induced apoptosis in ccRCC cells, which was further confirmed by increased activities of caspase-3 and caspase-9. Interestingly, we found that lncRNA-LET increased the level of Bax and decreased the level of Bcl-2. As the ratio of Bax/Bcl-2 contributes to the opening of mitochondrial permeability transition pore, it indicated that the induction of apoptosis by lncRNA-LET may be associated with mitochondria-mediated apoptosis. Therefore, the mitochondrial membrane potential and release of cytochrome *C* were detected. Our results demonstrated that lncRNA-LET modulated mitochondrial membrane potential and enhanced the release of cytochrome *C*, indicating that apoptosis of ccRCC induced by lncRNA-LET may belong to mitochondria-mediated apoptosis. Additionally, we found that lncRNA-LET increased the levels of DKK1 and TIMP2, which are involved in the regulation of cell migration [28–30], suggesting that lncRNA-LET may also have an effect on the metastasis of RCC. More researches are needed to verify this speculation.

Furthermore, how lncRNA-LET performed its tumor-suppressor role in ccRCC was explored. Li et al. revealed that miR-373-3p promoted tumorigenesis of RCC in vitro and in vivo [22]. In our study, we identified lncRNA-LET as a direct target of miR-373-3p. Interestingly, we found that the levels of DKK1 and TIMP2, which are two verified targets of miR-373-3p, were increased by lncRNA-LET. These results prompt us that lncRNA-LET may also modulate the expression of miR-373-3p target genes. Thus, rescue experiments were performed in our study. The results showed that miR-373-3p down-regulated the lncRNA-LET-induced increase of DKK1 and TIMP2 levels, and reversed the effects of lncRNA-LET on cell cycle and apoptosis. These results indicate that lncRNA-LET may perform its tumor-suppressor role in ccRCC cells through regulating the expression of miR-373-3p target genes. In 2011, Salmena et al. proposed a concept of competing endogenous RNA (ceRNA) [31] that targets of microRNA, such as mRNAs, lncRNAs and pseudogenes, can inversely target microRNAs using their microRNA response elements, thus modulating the expression of target genes and resulting in their various roles. We hypothesize that lncRNA-LET may act as a tumor-suppressor in ccRCC through a ceRNA pattern. As the ceRNA network is a large-scale regulatory network, there must be other microRNAs which lncRNA-LET may target to perform its role in ccRCC. However, our study focused on only miR-373-3p, there remains a large scale of microRNAs which can be targeted by lncRNA-LET. Hence, further explorations are needed to reveal the mechanism underlying lncRNA-LET.

## Conclusions

In the present study, lncRNA-LET repressed cell cycle, induced apoptosis and inhibited tumor growth of ccRCC by targeting miR-373-3p. We identified lncRNA-LET as a tumor-suppressor in ccRCC. The results of the present study provide a potential biomarker and therapeutic target for ccRCC treatment.

## Data Availability

The datasets used and analyzed during the current study are available from the corresponding author on reasonable request.

## Abbreviations

ccRCC: clear cell renal cell carcinoma
lncRNA: long non-coding RNA
DKK1: Dickkopf-1
TIMP2: tissue inhibitor of metalloproteinase-2
FBS: fetal bovine serum
NC: negative control
EV: empty control
PI: propidium iodide
